# 

**Published:** 1904-05

**Authors:** 


### CONTENTS.

ORIGINAL COMMUNICATIONS—

NOTES OF A TRIP TO NEW YORK, PHILADELPHIA AND RICHMOND. By James McFadden Gaston, M D., Atlanta, Ga. .......... 73

HEADACHE. By Chas. E. Boynton, M.D., Atlanta, Ga. .......... 84

PNEUMONIA AND ITS TREATMENT. By Henry R. Slack, Ph.M., M.D., LaGrange, Ga. .......... 94

CHRONIC CATARRH OF THE STOMACH. By J. N. LeConte, M.D., Atlanta, Ga. .......... 99

DISPLACEMENTS. By S. T. Barnett, M.D., Atlanta, Ga. .......... 106

SOCIETY REPORTS—

FLORIDA MEDICAL ASSOCIATION .......... 111

EDITORIAL NOTES AND COMMENTS—

MEETING OF THE MEDICAL ASSOCIATION OF GEORGIA .......... 113

NEWS AND NOTES .......... 120

CONTENTS CONTINUED .......... X

BOOK REVIEWS—

Polk's Medical Register and Directory of North America.................... 123

SELECTIONS AND ABSTRACTS—

The Transmission of Diphtheria.................................... 124

Treatment of Hip-Joint Disease.................................... 132

The Bicycle and Crime.................................... 137

Chronic Nasal Catarrh—A Simple and Effective Treatment.................... 140

Supreme Court State of New York.................................... 142

When Your Case is Weak Abuse the Other Side.................................... 144

MEDICAL ITEMS—CONTINUED........................ xviii, xx, xxii, xxiv, xxvi, xxviii.

